# Side of Limb-Onset Predicts Laterality of Gray Matter Loss in Amyotrophic Lateral Sclerosis

**DOI:** 10.1155/2014/473250

**Published:** 2014-07-01

**Authors:** Qiuli Zhang, Cuiping Mao, Jiaoting Jin, Chen Niu, Lijun Bai, Jingxia Dang, Ming Zhang

**Affiliations:** ^1^Department of Medical Imaging, First Affiliated Hospital, Medical College Xi'an Jiaotong University, 277 West Yanta Road, Xi'an, Shaanxi 710061, China; ^2^Department of Neurology, First Affiliated Hospital Medical College Xi'an Jiaotong University, 277 West Yanta Road, Xi'an, Shaanxi 710061, China; ^3^The Key Laboratory of Biomedical Information Engineering, Ministry of Education and Department of Biomedical Engineering, School of Life Science and Technology, Xi'an Jiaotong University, No. 28, Xianning West Road, Xi'an, Shaanxi 710049, China

## Abstract

Conflicting findings have been reported regarding the lateralized brain abnormality in patients with amyotrophic lateral sclerosis (ALS). In this study, we aimed to investigate the probable lateralization of gray matter (GM) atrophy in ALS patients. We focused on the relationship between the asymmetry in decreased GM volume and the side of disease onset in patients with limb-onset. Structural imaging evaluation of normalized atrophy (SIENAX) and voxel-based morphometry (VBM) were used to assess differences in global and local brain regions in patients with heterogeneous body onset and subgroups with different side of limb-onset. We found global brain atrophy and GM losses in the frontal and parietal areas in each patient group as well as left predominant GM losses in the total cohort. The intriguing findings in subgroup analyses demonstrated that the motor cortex in the contralateral hemisphere of the initially involved limb was most affected. We also found that regional brain atrophy was related to disease progression rate. Our observations suggested that side of limb-onset can predict laterality of GM loss in ALS patients and disease progression correlates with the extent of cortical abnormality.

## 1. Introduction

Amyotrophic lateral sclerosis (ALS) is a fatal disease characterized by the degeneration of upper and lower motor neurons, leading to hyperreflexia, spasticity, muscle weakness, and fasciculation [1]. Increasing evidence from clinical, pathological, and genetic studies has characterized ALS as a multisystem degeneration on the continuum with frontotemporal dementia (FTD) [1, 2]. The high prevalence of cognitive and behavioral impairments, even at times as severe as frank dementia, is now considered as another crucial feature in ALS [2–4].

The application of advanced neuroimaging techniques in detecting cortical abnormality has revealed compelling findings that match well with clinical manifestations and pathological abnormalities in ALS [5, 6]. For instance, the findings of focal morphological changes within the motor homunculus are consistent with the nature of focal onset and motor phenotype heterogeneity in ALS [7, 8]. Additionally, when comparing patients with pure motor syndromes with those with cognition or behavior dysfunction, the concurrent atrophy in motor cortex and frontal and/or temporal regions provides convincing insight into the relation between clinical profiles and the underlying pathological impairments* in vivo* [9–11].

However, inconsistent findings have also been reported in ALS [6]. Amongst these, the important and common finding of asymmetry or unilateral predominant structural and functional abnormality has not been comprehensively investigated. First, asymmetrical symptom onset is a well-established clinical feature in early stage of ALS, which may imply a lateralized brain pathology [12, 13]. Furthermore, intra- and inter-hemisphere asymmetry as a distinctive feature for differential diagnosis in other neurodegenerative diseases and subtypes of FTD [14–16], together with the finding that ALS and FTD may share similar degeneration pattern [17, 18], point to the fact that asymmetry may contribute to the heterogeneity of clinical presentations in ALS as well. Emerging evidence has suggested that the function of the frontal lobe, which was often involved in ALS, is divergent among hemispheres of the brain [19–22]. Therefore, on the basis of “prion-like” or network-based propagation [14, 23–25], the involvement of specific frontal areas adjacent to or functional linked with motor cortex in different hemisphere could be associated with divergent clinical profiles. For example, damage to the left hemisphere would prone to induce language deficits [26] while damage to the right hemisphere may produce attention or emotional disturbances along with disease progression [19, 20], all of which were observed clinically in the diverse ALS population [4, 18, 27]. Therefore, the existence of asymmetrical brain pathology may be the key for understanding the widely heterogeneous clinical features, as the case in FTD.

Nevertheless, lateralized structural changes are not thought to be a notable aspect in ALS [6, 28–30]. In addition, inconsistent reports on the unilateral predominated brain atrophy also skew our understanding of the neurological impairments in ALS. Specifically, some studies concluded rightward degeneration based on pronounced gray matter (GM) loss or functional abnormality in the right hemisphere [31–35]. While other studies confronted the unilateral predominant atrophy as a separate clinical constellation or a specific group effect [36–41].

Among these, only a few studies have examined the asymmetry issue by measuring the relationship between lateralized brain metabolic ratio changes and clinical presentations or separating patients based on the laterality of limb-onset. These studies suggested that the lateralization of clinical syndromes mostly corresponds to brain abnormalities in the contralateral hemisphere [39–41]. However, the contralateral hemisphere of the lateralized clinical presentation or side of syndrome defect was unclear, because the variable spreading pattern [42] and diminished asymmetry of clinical presentation as the disease progresses will make the lateralized clinical presentation in these former studies [39, 40] differ with the laterality of limb-onset in later study [41]. Therefore, combining with the correlation between region of onset and the characteristics of neuron loss in the lower motor neurons [43], it is critical to define the lateralization of syndrome according to the sidedness of onset, as suggested by Zhang et al. [41].

Here we aimed to explore lateralization or asymmetrical GM loss in ALS patients and establish the relationship between the sides of limb defect at disease onset with imaging findings to help clarify the neurologic basis of the various motor syndromes in ALS. Optimized and reliable voxel-based morphometry (VBM) and Structural imaging evaluation of normalized atrophy (SIENAX) were used in the following analyses to identify regional and gross changes. First, all patients with heterogeneous body part defects at disease onset were compared with normal controls to assess whether there is a unilateral GM abnormality. Then subgroup analyses were conducted on patients with unilateral homogenous side of limb-onset with their respective normal controls to investigate for GM loss patterns in each group. Based on the previous findings [7, 8, 13, 41, 43], we hypothesize that amongst patients with homogenous side of disease onset, the prominent GM abnormality will be located primarily in the contralateral hemisphere to the initially involved limbs.

## 2. Materials and Methods

### 2.1. Subjects

Forty-three patients (27 males, 32 to 71 years old) from October 2011 to August 2013 with either a definite or probable diagnosis of sporadic ALS, according to the revised EI Escorial criteria, were enrolled in this study [30]. Based on the body part affected at disease onset, 36 patients were defined as limb-onset and 7 patients were bulbar-onset. Of the 36 patients with limb-onset, 20 patients presented with right side syndromes at onset (16 upper extremity, 4 lower extremity), and 16 patients present with left limb involvement (12 upper extremity, 4 lower extremity). Disease severity (full score = 40) and disease progression (40-ALSFRS/disease duration (months)) were assessed with the ALS functional rating scale (ALSFRS) [31]. In addition, 43 age- and gender-matched control subjects (27 males, ranged 30–74 years) were recruited. To minimize confounding factors caused by non-ALS-related alterations of the brain, patients with current or history of any of the listed conditions below were excluded (brain injury, trauma, epilepsy, vascular disease, psychiatric illness, or other systemic diseases). All 86 patients enrolled were right handed and had a Mini-Mental State Exam (MMSE) score of ≥26 [32]. Written consent was obtained for all subjects and this study was approved by the Research Ethics Committee of the First Affiliated Hospital of Medical College of Xi'an Jiaotong University.

### 2.2. Study Design

Composite study population included 43 ALS patients and 43 normal controls. Subgroup analyses were composed of (1) twenty patients with right limb-onset and their age- and gender-matched healthy controls and (2) sixteen patients with left limb-onset and their age- and gender-matched healthy controls. The seven patients with bulbar-onset were not included in subgroup analyses due to small sample size and potential reduction in statistical power.

### 2.3. Imaging Acquisition

All imaging data were acquired using a 3T MR system (General Electric, Signal HDxt, Milwaukee, WI, USA) equipped with an eight-channel head-coil; a high resolution T_1_-weighted image was acquired for global and local brain regions measurements. Acquisition parameters are as follows: 3D T_1_ fast spoiled gradient echo sequence; repetition time (TR) = 10.8 ms; echo time (TE) = 4.8 ms; slice orientation, parallel with the anterior and posterior commissure line; matrix = 256 × 256; field of view = 256 mm × 256 mm; slice thickness = 1 mm; no gap; and 140 continuous slices were acquired, covering the whole brain. Routine T_2_ weighted images, implemented with propeller sequences, are as follows: TR = 4680 ms; TE = 105.2 ms; field of view = 256 mm × 256 mm; slice thickness = 5 mm. T_1_ weighted images are as follows: TR = 2928 ms; TE = 22 ms; slice thickness = 5 mm. Routine T_1_ and T_2_ weighted images were used to get rid of subjects with obvious cerebral disease such as stroke or severe infarction. The scan lasted for approximately 8 minutes.

### 2.4. Statistical Analysis

Statistical analysis was performed with the Statistical Package for Social Sciences for Windows (Version 13; SPSS, Chicago, Illinois). The normality of data was statistically examined by using the Kolmogorov-Smirnoff tests. Group differences of demographic characteristics including age, gender, and clinical measurements, as well as tissue specific volume calculated by SIENAX, were tested by using independent two-sample Student's *t*-test, chi-square test, and analysis of covariance, as appropriate.

Parametric or nonparametric correlations, as well as partial correlation analyses, were performed to assess the relationship between volume of specific tissue-type and other MR imaging and clinical measurements, as appropriate.

### 2.5. Global Brain Measurements

Normalized global and tissue-type volume for head size were calculated with cross-sectional software tool—SIENAX, part of FSL [44, 45] (Version 2.6; FMRIB software library, http://fsl.fmrib.ox.ac.uk/fsl/fslwiki/SIENA). More precisely, extracted brain from single subject's whole-head input data was estimated by removing skull images [46] and was then used to affine-registered to MNI152 space [47, 48]. Subsequently, the volumetric scaling factor determined by the skull image in registration step was used to assess normalized tissue-specific volume, after tissue segmentation with partial volume estimation using FAST4 [49]. Consequently, for each subject, normalized volumes of the GM volume (NGMV), neocortical volume (NCV), and WM volume (NWMV) were obtained. Brain parenchyma fraction (BPF) was also assessed.

### 2.6. Regional Brain Atrophy Evaluation

Regional GM comparisons between all the groups were assessed using the optimized VBM tools, FSL-VBM (http://fsl.fmrib.ox.ac.uk/fsl/fslwiki/FSLVBM) [50, 51], from the FMRIB Software Library. Firstly, for each individual dataset, brain extracted tools were used to remove nonbrain tissues [46]. The FAST4 was then carried out for tissue-type segmentation [49]. The resulting GM partial volume images were aligned to MNI152 standard space using FLIRT [47, 48], followed optionally by nonlinear registration using FNIRT [52, 53]. A left-right symmetric study-specific GM template was averaged from the total number of subjects' gray-matter-segmented native images, both in whole group and subgroups analyses, which were then nonlinearly reregistered. In order to correct for local expansion or contraction, the registered partial volume images were modulated by dividing by the Jacobian of the warp field. The modulated segmented images were finally smoothed with an isotropic Gaussian kernel with a sigma of 3 mm.

Differences in the distribution of GM between patients and controls were examined by using permutation-based nonparametric testing, correcting for multiple comparisons by implementing threshold-free cluster enhancement (TFCE) [54].

In the total and subgroup analyses, age and total brain volume (the sum of NGMV, NWMV, and normalized cerebrospinal fluid) were used as nonexplanatory covariates in the general linear model.

The significance threshold was set at *P* < 0.05 (family-wise error (FWE) corrected) for whole group analysis. In subgroup analysis, statistical map was derived from an uncorrected voxel level threshold at *P* < 0.001, which did not survive after FWE correction. Only clusters comprising more than 20 adjacent voxels were included.

### 2.7. Correlation Analysis

In order to further evaluate the clinical relevance in whole brain tissue-specific measurements and regional GM density, we performed correlation analyses. Regional GM density was extracted over the clusters identified in the groups' analyses, according to its anatomical components. Clinical variables mainly include disease severity assessment—ALSFRS, disease duration, and disease progression rate.

## 3. Results

### 3.1. Demographic, Clinical Characteristics

In whole group analysis, there were no significant differences in age, gender, and MMSE score between 43 patients with sporadic ALS and 43 healthy controls (Table 1). In subgroup analysis, although age, gender, and MMSE score did not differ in patients with right and left limb-onset compared with controls, patients with right limb-onset were significantly older and presented with a less ALSFRS score (Table 2).

### 3.2. Normalized Global and Tissue-Specific Measurements

**Figure 1:**
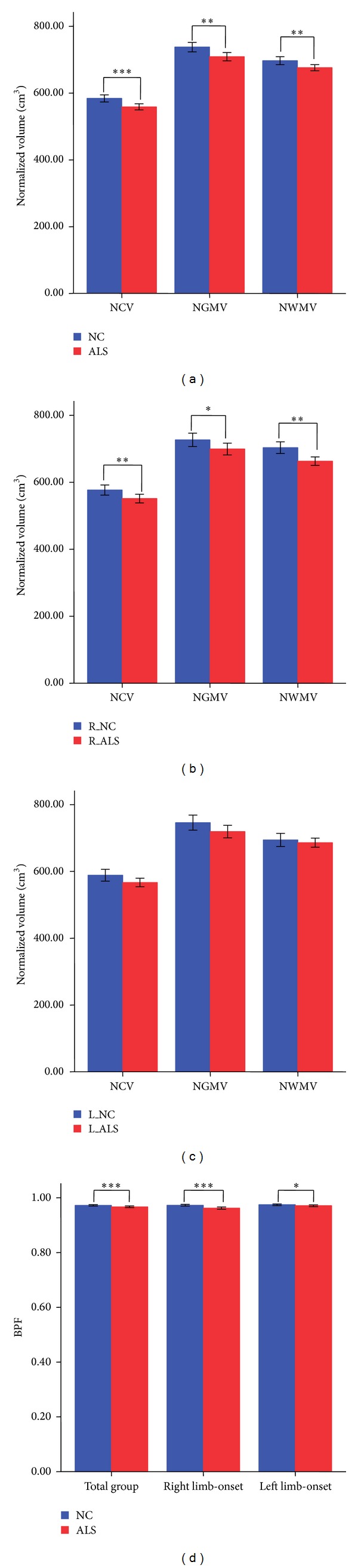
Global volumetric measurements comparisons in total group ((a), (d)) and subgroups between ALS patients with right limb-onset ((b), (d)), patients with left limb-onset ((c), (d)), and corresponding normal controls. ***P < 0.001; **P < 0.01; *P < 0.05.

Because the normalized volume of tissue-specific measurements was strongly age-related, the *r* values between age and global measurements range from −0.259 for NWMV to −0.589 for BPF (all of the correlations were statistically significant at *P* < 0.05). We compared group differences both before and after adjusting with age. In whole group analysis, we found reduced NCV, NGMV, NWMV, and BPF in patients with ALS (Table 3 and Figure 1). In subgroup analysis, significant group differences including NGMV, NWMV, NCV, and BPF were found between patients with right limb-onset and normal controls (Table 4 and Figure 1). In patients with left limb-onset, only BPF was found statistically smaller than those in normal controls after correcting age (Table 4 and Figure 1).

### 3.3. Regional GM Losses in Total and Subgroup Analysis

**Figure 2:**
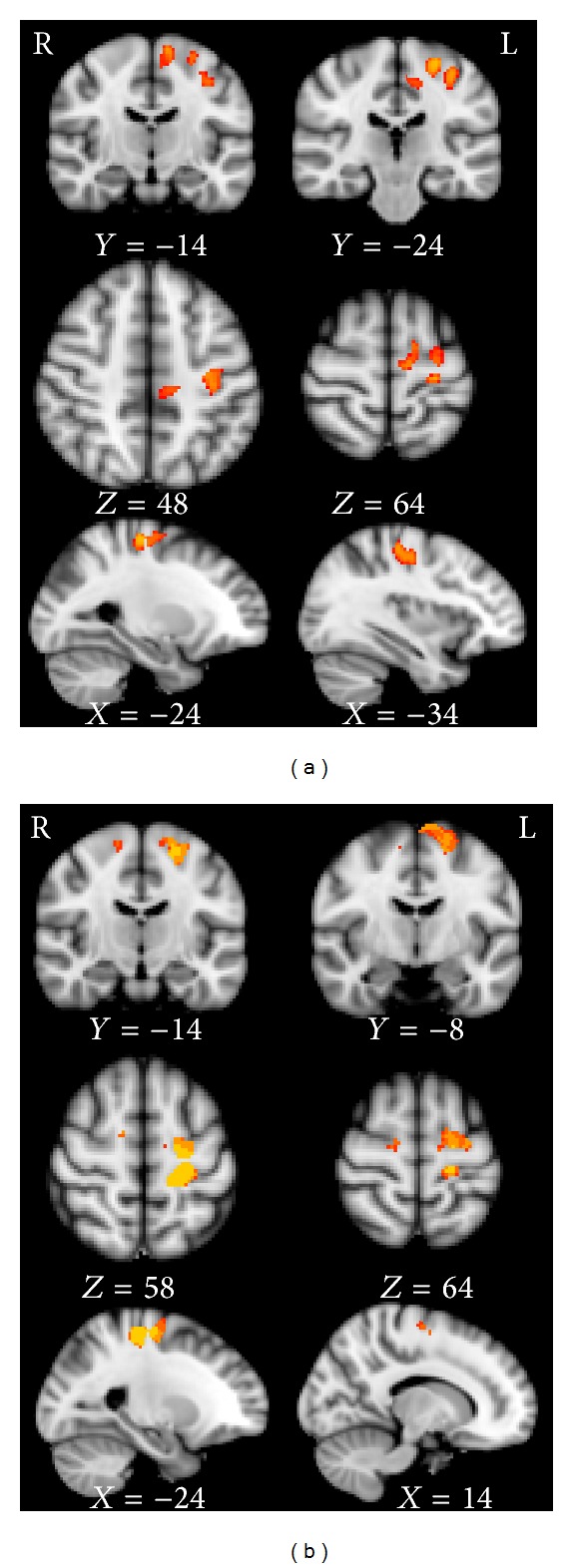
(a) GM losses in total group analysis between ALS patients and normal controls (*P* < 0.05, FWE-corrected). (b) GM losses in subgroup analysis between ALS patients with right limb-onset and corresponding normal controls (*P* < 0.001, uncorrected).

In total group analysis, prominent GM volume reductions were found in the left frontal and parietal cortices including the precentral gyri, superior frontal gyri, supplementary motor areas, and postcentral gyri (*P* < 0.05, FWE corrected) (Table 5 and Figure 2).

**Figure 3:**
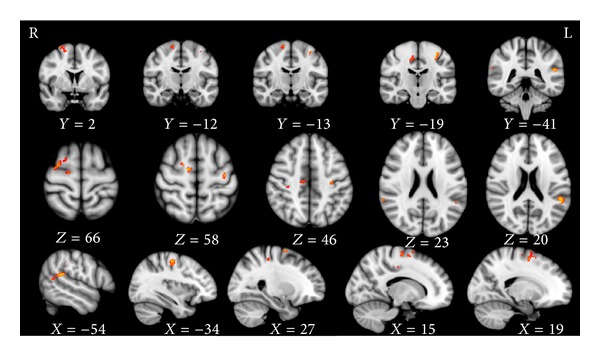
GM losses in subgroup analysis between ALS patients with left limb-onset and corresponding normal controls (*P* < 0.001, uncorrected).

In subgroup comparisons, patients with right limb-onset showed decreased GM volume in the bilateral precentral gyri, left superior frontal gyri, supplementary motor areas, and postcentral gyri compared with normal controls (*P* < 0.001, uncorrected) (Table 6 and Figure 2). GM losses in patients with left limb-onset were mainly located in the bilateral precentral gyri, right superior frontal gyri, supplementary motor areas, postcentral gyri, bilateral supramarginal gyri, parietal operculum, and angular gyri in the left side (*P* < 0.001, uncorrected) (Table 7 and Figure 3).

### 3.4. Relationships between Neuroimaging and Clinical Outcomes in Whole Group and Subgroup Patients

No statistically significant relations were found between normalized global measurements and clinical profiles, both in whole group and subgroup patients before and after correcting for age.

**Figure 4:**
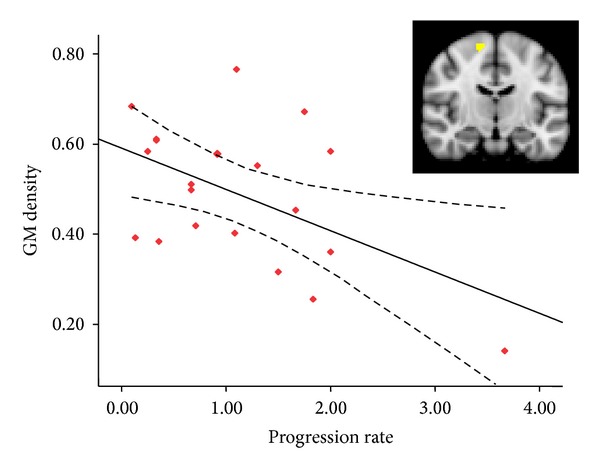
GM density in the right precentral gyri showed a negative correlation with disease progression rate (*r* = −0.505, *P* = 0.020).

Correlation analyses in the whole group comparison found a positive correlation between the GM density in the left postcentral gyri and disease severity score—ALSFRS (*r* = 0.38, *P* = 0.012). In patients with right limb-onset, we found that disease progression rate was negatively related with GM density in the right precentral gyri (*r* = −0.515, *P* = 0.020) (Figure 4). There was no statistical significant relationship between clinical features of patients and neuroimaging features in patients with left limb-onset.

## 4. Discussion

In this study, we found global and local brain region atrophy in patients with ALS. Additionally, BPF was a sensitive biomarker in assessing global brain atrophy. Regional brain atrophy profile in each patient group demonstrated that GM loss was primarily but not exclusively in the primary motor, premotor, and supplementary motor areas in the frontal lobes and associated with variable parietal lobes involvement. Interestingly, unilateral dominant GM losses in patients with heterogeneous body-onset in total group were mainly determined by patients with right limb-onset. Furthermore, subgroup analyses have implied that motor cortex in the contralateral hemisphere of the initially involved limb was most affected. Lastly, compared with left limb-onset patients, the age at disease onset were much older in patients with right limb-onset, who also presented with more severe disease disability.

### 4.1. Demographics and Clinical Findings

In contrast to left limb-onset patients, older age and lower ALSFRS were found in patients with right limb-onset (*P* = 0.005, 0.043, resp.). To verify whether the difference of age was caused by the discrepancy of age at disease onset or derived from variable disease durations (see Table 2), we further compared age at disease onset between the two subgroups. Interestingly, we did find that patients with left limb-onset were much younger at disease onset in our study compared to those with right limb-onset (Table 2, *P* = 0.008). Additionally, patients with right limb-onset who were significantly older had lower ALSFRS scores indicating that a more advanced disease severity is potentially linked with age, a negative predictor for prognosis [44]. However, the questionnaire of ALSFRS in our study and widely used revised-ALSFRS are insufficient to make appropriate and advisable assessing of functional disability caused by nondominant hand [13, 55]. This is particularly relevant since more than half of the patients presented with upper limb dysfunction at disease onset in right and left limb-onset subgroups (16/20, 12/16, resp.). Therefore, we thought it was not adequate to speculate that the ALSFRS in patients with left limb was truly higher because of biased functional disability assessments in our right-hand dominant patients' cohorts. However, even though our results were consistent with Rule et al. regarding the differences in the mean age and ALSFRS-*R* score between patients with right and left side of body impairments, which was not statistically significant [38], this interesting inference should be further verified with a larger sample of patients.

### 4.2. Global Atrophy in ALS

Global brain atrophy in ALS patients, as indicated in our study with reduced NCV, NGMV, NWMV, and BPF, had been revealed in several previous studies [11, 32, 36, 56, 57]. Amongst these, decreased BPF can be found even in the absence of obvious brain parenchyma volume loss suggesting that BPF may be a more sensitive biomarker than other measurements in assessing global brain atrophy [32, 58]. This in fact proved to be the case in our study in which the subgroup comparison only showed decreased BPF in patients with left limb-onset, all of whom did not show remarkable gray or white matter volume loss. Consistent with the obvious regional GM loss in later studies, there were reduced NCV and NGMV. Unlike the global measurements mentioned above, decreased NWMV had been found in some studies [56, 57], but not others [11, 32]. We speculated that this discrepancy is the result of the highly heterogeneous study cohorts and methodological sensitivities. When using more advanced methods such as in Rajagopalan et al., studies indeed confirmed white matter abnormality in ALS [59]. The differences between global indexes in patients with right and left limb-onset implied that global atrophy was more prevalent in older patients when compared with age-matched healthy controls. In addition, this is partially relevant with the context of our findings that global brain atrophy measurements are strongly age-related, as well as the hypothesis suggested by Mezzapesa et al. that ALS pathology enhanced normal aging [32].

### 4.3. Regional and Lateralized GM Loss in Whole and Subgroup Analysis

We found regional brain atrophy of the motor cortex particularly in the frontal lobe and postcentral gyri, supramarginal gyri, angular gyri, and parietal operculum in the parietal lobe, all consistent with previous findings [9, 11, 21, 22, 60–63]. All of these regions participated in motor performance or motor imagery [64] and GM losses in these regions reflected dysfunctions of both motor performance and motor imagery in ALS [37]. We suggested that atrophy in premotor and supplementary motor areas in each group extended the feature of focal onset in primary motor cortex to other motor related areas in the frontal areas [7, 8]. These findings and additional GM abnormalities in parietal areas are consistent with the longitudinal findings performed by Verstraete et al, suggesting that neurodegeneration begins with primary motor cortex and extends to secondary motor cortex in the frontal and parietal lobes [63]. However, in our subgroup analysis, we uncovered an intriguing pattern that the more extensive GM losses were found in patients with left limb-onset compared to those in patients with right limb-onset. This finding may be due to the fact that general widespread GM abnormalities are often associated with greater disease disability, but it certainly warrants further investigation to clarify whether disease disability assessed by ALSFRS in patients with left limb-onset was artificially higher as we discussed above. Another possibility that deserves further study is that patients with left limb-onset may represent the ALS patients who were younger than 45 years old at the time of disease onset and characterized in prevalent upper motor neuron involvements, thus, leading to widespread GM abnormalities [65]. These profound differences between the right limb and left limb-onset subgroups in their clinical features and GM losses are intriguing and require further exploration.

Except for the aforementioned regional anatomical abnormal, the main finding in our study was that motor cortex in the contralateral hemisphere of the initially involved limb was the most heavily affected region. This was consistent with and supplemented the findings in Zhang et al. [41] and the pathological findings [13]. In addition, according to the pattern of GM loss in total cohorts and patients with right limb-onset, we suggested that GM loss in the whole group population was mainly reflective of the abnormalities caused by the subpopulation of patients with right limb-onset. These findings indicated to us that it was noteworthy to take clinical profiles into account when interpreting cortical abnormality in group level, especially in those with heterogeneous backgrounds. Furthermore, the macroscopic signatures of GM atrophy in subgroup comparisons suggested that, when comparing with patients with left limb-onset, right limb-onset is more likely to have language deficits which was as common as executive dysfunction in ALS [27]. This may be a consequence of the susceptibility of Broca's areas (Brodmann areas 44, 45) in the left inferior frontal cortex to prion-like contiguous spread from the motor cortex or through network-based structural white matter connections [23, 26]. This pathological basis associated with more common right hand deficits [66] may explain the high prevalence of language deficits as well as the proved high sensitivity of the verbal frequency in assessing executive deficits in ALS [27]. Meanwhile, among patients with left limb-onset, abnormal GM in the right motor cortex may be easily accompanied with more extensive frontal impairments in the ipsilateral side and may induce behavioral dysfunction [14, 15]. Additionally, the proportion of cognitive and/or behavioral impairments was strongly associated with the different progression rates among individuals, which may mean that patients with fast disease progression may be more prone to show cognition or behavioral dysfunction in cross-sectional study and vice versa. But these inferences need to be confirmed by the cognitive screening and longitudinal evaluations.

In addition, one must keep in mind that the unilateral predominant cortical abnormality determined by the side of limb-onset does not tell the whole story. Along with the temporal evolution of disease, ALS does eventually involve other limbs symmetrically and the presenting of GM loss in the ipsilateral hemisphere of the initially involved limb may reduce this asymmetry, as indicated in our subgroup analysis. More than 18/20 and 9/16 of patients presented with contralateral upper and/or lower limb dysfunction in patients with right and left limb-onset, respectively. Moreover, the association of cognitive or behavioral impairments in ALS and their variability [4, 15], as well as heterogeneous disease progression rate in individual ALS patients, will increase or reduce the asymmetry dominated by motor symptoms and add to the complexity of this disease.

### 4.4. Correlation Analysis between Clinical Variables and MR Findings

In the whole group comparisons, we found a positive correlation between the GM density in the left postcentral gyri and ALSFRS. We inferred that such correlation would be found in a less heterogeneous patient group with right limb-onset because the differences in whole group comparisons were mainly derived from disease related effects in this group. Nevertheless, congruous with other studies, this relation was only found in pooled patients with different clinical profiles [7, 8].

Meanwhile, a negative relationship between GM density in the right precentral gyri and disease progression rate was found in patients with right limb-onset. Warren et al. have hypothesized that the involvement of longer-range connections corresponds with rapid spread in contrast to slower spread caused by involvement of clustered connections [14]. Compared with ipsilateral frontal and parietal areas adjacent to precentral gyri, GM losses in the contralateral hemisphere were relatively remote areas and represented rapid disease progression. In previous studies, it has been suggested that the involvement of the temporal lobe can be a marker for more rapid disease progression [28]. In addition, several longitudinal studies have implied that, compared with the disassociation between rapidly progressive clinical profiles and less or nonprogressive deterioration of primary motor cortex, the extensive involvements of motor function or cognitive, behavioral related brain regions were remarkable in the follow-up [28, 67]. In sum, we proposed that the correlation between GM density in the right precentral gyri and disease progression rate indicated that the relatively distant region from the initial onset regions—the left precentral gyri in patients with right limb-onset in our study—was involved later when disease progressed. Thus, along with temporal evolution of the disease, faster disease progression will present with more areas involved and relate with the extent of cortical abnormality in the disease course. This finding may present a novel hypothesis to explain the changing pattern of remote brain regions involvement with disease progression rate as described in previous findings. In addition, we propose that the lack of relation between abnormal brain regions and clinical variables in patients with left limb-onset might be caused by inadequate disease disability assessments that would also result in a less accurate disease progression rate.

### 4.5. Limitations

There are several limitations in this study. Firstly, patients with bulbar-onset were not included in the subgroups and this therefore made it impossible to evaluate the distinguished features of brain abnormal in these patients. Secondly, several studies confirm that ALS patients with large scales of cognitive and behavioral impairments were associated with different vulnerability of specific genetic mutations that were not considered in this study. Lastly, lack of fully exploring of cognitive domains and mutant genetic screening in our patients is fundamental deficit and limited us and made us unable to further clarify the brain atrophy profiles and asymmetries.

## 5. Conclusions

In this study, we found global and regional brain atrophy in patients with ALS. The topographic characteristics of subgroup analysis further indicated that the motor cortex in the contralateral hemisphere of the initially involved limb was most affected with relatively sparing of the ipsilateral brain. The unilateral dominant topography of brain changes implied that the clinical profiles of patients with different side of limb-onset might become divergent, particularly focusing on the extramotor syndromes, as disease progresses. The unexpected findings of demographic and clinical discrepancy between patients with right or left limb-onset also highlight a novel distinct feature of ALS, which deserves further exploration.

## Figures and Tables

**Table 1:** Summary of clinical characteristics between patients with ALS and normal controls in whole group analysis.

	NC (*n* = 43)	ALS (*n* = 43)	*P*
Age	51.8 ± 9.4	53.5 ± 9.0	0.389
Male, *n*	26	26	1
MMSE	28.8 ± 1.1	28.2 ± 1.9	0.054
ALSFRS	n/a	30.1 ± 6.6	—
Disease duration (months)	n/a	16.9 ± 16.5	—
Disease progression rate	n/a	0.98 ± 1.03	—

NC: normal controls; MMSE: Mini-Mental Status Evaluation; disease progression rate = (40-ALSFRS)/disease duration. n/a: not applicable.

**Table 2:** Summary of clinical characteristics between patients with ALS and normal controls in subgroups.

	R_limb-onset (*n* = 20)	R_NC (*n* = 20)	*P* ^1^	L_limb-onset (*n* = 16)	L_NC (*n* = 16)	*P* ^2^	*P* ^3^
Age	57.5 ± 8.1 (55.8 ± 8.0)^a^	56.0 ± 6.4	0.520	48.4 + 9.7 (47.4 ± 9.9)^a^	50.6 ± 9.0	0.515	0.005 0.008^a^
Male, *n*	14	14	1	9	9	1	0.307
MMSE	27.5 ± 2.1	28.8 ± 1.2	0.051	28.9 ± 1.6	29.2 ± 1.0	0.508	0.055
ALSFRS	27.7 ± 6.6	n/a	—	32.3 ± 6.3	n/a	—	0.043
Disease duration (months)	20.9 ± 20.8	n/a	—	13.8 ± 6.9	n/a	—	0.239
Disease progression rate	1.12 ± 0.88	n/a	—	0.66 ± 0.62	n/a	—	0.088

R_NC: normal controls corresponding to patients with right limb-onset; L_NC: normal controls corresponding to patients with left limb-onset. ^a^Mean age of disease onset in patients with right and left limb-onset and corresponding *P* value.

*P* ^1^ represents the statistical differences in ALS patients with right limb-onset and corresponding normal controls. *P* ^2^ represents the statistical differences in ALS patients with left limb-onset and corresponding normal controls. *P* ^3^ represents the statistical differences in subgroups between ALS patients with right and left limb-onset.

**Table 3:** Global-brain volumetric measurements in total group of patients with ALS and normal controls by SIENAX.

	NC	ALS	*P* ^1^	*P* ^2^
BPF	0.97 ± 0.01	0.96 ± 0.01	0.009	0.000
NCV (mm^3^)	584.0 ± 34.6	558.7 ± 30.2	0.001	0.000
NGMV (mm^3^)	737.7 ± 46.4	699.4 ± 105.9	0.016	0.004
NWMV (mm^3^)	697.0 ± 38.2	675.9 ± 30.1	0.006	0.004

BPF: brain parenchyma fraction; NCV: normalized neocortex volume; NGMV: normalized gray matter volume; NWMV: normalized white matter volume.

*P* ^1^: *P* value in total group analysis between patients with ALS and normal controls, by using two-sample *t*-test.

*P* ^2^: *P* value in total group analysis between patients with ALS and normal controls, by using analysis of covariance, with age as covariate.

**Table 4:** Global-brain volumetric measurements in ALS patients with right or left limb-onset and respective normal controls by SIENAX.

	R_NC	R_limb-onset	*P* ^1^	L_NC	L_limb-onset	*P* ^2^
BPF	0.97 ± 0.007	0.96 ± 0.009	0.000	0.974 ± 0.006	0.971 ± 0.005	0.016
NCV (mm^3^)	576.90 ± 34.04	551.37 ± 28.93	0.008	588.46 ± 35.43	566.68 ± 25.80	0.053
NGMV (mm^3^)	726.61 ± 44.80	699.30 ± 39.59	0.042	746.02 ± 45.15	680.55 ± 167.20	0.113
NWMV (mm^3^)	703.38 ± 38.84	663.01 ± 28.44	0.001	694.24 ± 39.67	685.97 ± 27.15	0.402

*P* ^1^, *P* ^2^: *P* value in subgroup analysis between patients with right or left limb-onset and respective normal controls, by using analysis of covariance, adjusted for age.

**Table 5:** Brain areas with reduced GM volume in total group between ALS patients and normal controls (*P* < 0.05, FWE-corrected).

Brain anatomical regions	BA region	MNI coordinates of peak voxels (mm)			Cluster size	*T* value	*P* value
		*X*	*Y*	*Z*			
L_precentral gyri	4	−26	−24	62	450	4.85	0.0132
		−24	−22	58		4.80	0.0136
L_superior frontal gyri	6	−8	−8	72	124	4.79	0.0144
L_supplementary motor areas	6	−8	−10	70	28	4.50	0.015
L_postcentral gyri	3	−36	−26	54	60	4.25	0.0196

L: left; BA: Brodmann areas.

**Table 6:** Brain areas with reduced GM volume in ALS patients with right limb-onset and corresponding normal controls (*P* < 0.001, uncorrected).

Brain anatomical regions	BA region	MNI coordinates of peak voxels (mm)			Cluster size	*T* value	*P* value
		*X*	*Y*	*Z*			
L_precentral gyri	4, 6	−20	−30	58	337	4.56	0.0002
		−16	−30	60		4.52	
		−26	−26	58		4.42	
L_superior frontal gyri	6	−12	−8	70	208	4.55	0.0006
		−16	−6	68		4.49	
L_supplementary motor areas	6	−4	−8	72	20	3.22	0.0004
L_postcentral gyri	3	−20	−34	58	74	4.45	0.0002
R_precentral gyri	4	14	−12	62	48	3.59	0.0006

L: left; R: right.

**Table 7:** Brain areas with reduced GM volume in ALS patients with left limb-onset and corresponding normal controls (*P* < 0.001, uncorrected).

Brain anatomical regions	BA region	MNI coordinates of peak voxels (mm)			Cluster size	*T* value	*P* value
		*X*	*Y*	*Z*			
R_precentral gyri	4, 6	10	−18	48	89	4.07	0.0008
R_superior frontal gyri	6	14	−2	62	209	3.70	0.0008
R_supplementary motor areas	6	12	−6	58	28	3.42	0.0002
R_postcentral gyri	3	36	−28	42	33	4.09	0.0008
R_supramarginal gyri	40	62	−40	22	26	2.12	0.0002
L_precentral gyri	4, 6	−34	−14	54	111	3.56	0.0004
L_parietal opercular		−52	−38	20	28	3.92	0.0002
L_supramarginal gyri	40	−54	−46	18	93	3.42	0.0002
L_angular gyri	39	−44	−52	12	48	3.36	0.0004

L: left; R: right.

## References

[1] Robberecht W, Philips T (2013). The changing scene of amyotrophic lateral sclerosis. *Nature Reviews Neuroscience*.

[2] Turner MR, Hardiman O, Benatar M (2013). Controversies and priorities in amyotrophic lateral sclerosis. *The Lancet Neurology*.

[3] Byrne S, Elamin M, Bede P (2012). Cognitive and clinical characteristics of patients with amyotrophic lateral sclerosis carrying a C9orf72 repeat expansion: a population-based cohort study. *The Lancet Neurology*.

[4] Phukan J, Elamin M, Bede P (2012). The syndrome of cognitive impairment in amyotrophic lateral sclerosis: a population-based study. *Journal of Neurology, Neurosurgery & Psychiatry*.

[5] Turner MR, Agosta F, Bede P, Govind V, Lulé D, Verstraete E (2012). Neuroimaging in amyotrophic lateral sclerosis. *Biomarkers in Medicine*.

[6] Bede P, Hardiman O (2014). Lessons of ALS imaging: pitfalls and future directions—a critical review. *NeuroImage: Clinical*.

[7] Bede P, Bokde A, Elamin M (2013). Grey matter correlates of clinical variables in amyotrophic lateral sclerosis (ALS): a neuroimaging study of ALS motor phenotype heterogeneity and cortical focality. *Journal of Neurology, Neurosurgery and Psychiatry*.

[8] Schuster C, Kasper E, Machts J (2013). Focal thinning of the motor cortex mirrors clinical features of amyotrophic lateral sclerosis and their phenotypes: a neuroimaging study. *Journal of Neurology*.

[9] Irwin DJ, McMillan CT, Brettschneider J (2013). Cognitive decline and reduced survival in C9orf72 expansion frontotemporal degeneration and amyotrophic lateral sclerosis. *Journal of Neurology, Neurosurgery and Psychiatry*.

[10] Lillo P, Mioshi E, Burrell JR, Kiernan MC, Hodges JR, Hornberger M (2012). Grey and white matter changes across the amyotrophiclateral sclerosis-frontotemporal dementia continuum. *PLoS ONE*.

[11] Chang JL, Lomen-Hoerth C, Murphy J (2005). A voxel-based morphometry study of patterns of brain atrophy in ALS and ALS/FTLD. *Neurology*.

[12] Sabatelli M, Conte A, Zollino M (2013). Clinical and genetic heterogeneity of amyotrophic lateral sclerosis. *Clinical Genetics*.

[13] Mochizuki Y, Mizutani T, Takasu T (1995). Amyotrophic lateral sclerosis with marked neurological asymmetry: clinicopathological study. *Acta Neuropathologica*.

[14] Warren JD, Rohrer JD, Schott JM, Fox NC, Hardy J, Rossor MN (2013). Molecular nexopathies: a new paradigm of neurodegenerative disease. *Trends in Neurosciences*.

[15] Rohrer JD, Lashley T, Schott JM (2011). Clinical and neuroanatomical signatures of tissue pathology in frontotemporal lobar degeneration. *Brain*.

[16] Hornberger M, Wong S, Tan R (2012). In vivo and post-mortem memory circuit integrity in frontotemporal dementia and Alzheimer's disease. *Brain*.

[17] Ling SC, Polymenidou M, Cleveland DW (2013). Converging mechanisms in ALS and FTD: disrupted RNA and protein homeostasis. *Neuron*.

[18] Lillo P, Savage S, Mioshi E, Kiernan MC, Hodges JR (2012). Amyotrophic lateral sclerosis and frontotemporal dementia: a behavioural and cognitive continuum. *Amyotrophic Lateral Sclerosis*.

[19] Miller GA, Crocker LD, Spielberg JM, Infantolino ZP, Heller W (2013). Issues in localization of brain function: the case of lateralized frontal cortex in cognition, emotion, and psychopathology. *Frontiers in Integrative Neuroscience*.

[20] Iturria-Medina Y, Fernández AP, Morris DM (2011). Brain hemispheric structural efficiency and interconnectivity rightward asymmetry in human and nonhuman primates. *Cerebral Cortex*.

[21] Tsermentseli S, Leigh PN, Goldstein LH (2012). The anatomy of cognitive impairment in amyotrophic lateral sclerosis: more than frontal lobe dysfunction. *Cortex*.

[22] Trojsi F, Monsurr MR, Esposito F, Tedeschi G (2012). Widespread structural and functional connectivity changes in amyotrophic lateral sclerosis: insights from advanced neuroimaging research. *Neural Plasticity*.

[23] Polymenidou M, Cleveland DW (2011). The seeds of neurodegeneration: prion-like spreading in ALS. *Cell*.

[24] Kanouchi T, Ohkubo T, Yokota T (2012). Can regional spreading of amyotrophic lateral sclerosis motor symptoms be explained by prion-like propagation?. *Journal of Neurology, Neurosurgery and Psychiatry*.

[25] Seeley WW, Crawford RK, Zhou J, Miller BL, Greicius MD (2009). Neurodegenerative diseases target large-scale human brain networks. *Neuron*.

[26] Pulvermüller F, Fadiga L (2010). Active perception: sensorimotor circuits as a cortical basis for language. *Nature Reviews Neuroscience*.

[27] Taylor LJ, Brown RG, Tsermentseli S (2013). Is language impairment more common than executive dysfunction in amyotrophic lateral sclerosis?. *Journal of Neurology, Neurosurgery and Psychiatry*.

[28] Verstraete E, Veldink JH, Hendrikse J, Schelhaas HJ, van den Heuvel MP, van den Berg LH (2012). Structural MRI reveals cortical thinning in amyotrophic lateral sclerosis. *Journal of Neurology, Neurosurgery & Psychiatry*.

[29] Thivard L, Pradat P, Lehéricy S (2007). Diffusion tensor imaging and voxel based morphometry study in amyotrophic lateral sclerosis: relationships with motor disability. *Journal of Neurology, Neurosurgery and Psychiatry*.

[30] Grossman M, Anderson C, Khan A, Avants B, Elman L, McCluskey L (2008). Impaired action knowledge in amyotrophic lateral sclerosis. *Neurology*.

[31] Agosta F, Pagani E, Rocca MA (2007). Voxel-based morphometry study of brain volumetry and diffusivity in amyotrophic lateral sclerosis patients with mild disability. *Human Brain Mapping*.

[32] Mezzapesa DM, Ceccarelli A, Dicuonzo F (2007). Whole-brain and regional brain atrophy in amyotrophic lateral sclerosis. *American Journal of Neuroradiology*.

[33] Chen Z, Ma L (2010). Grey matter volume changes over the whole brain in amyotrophic lateral sclerosis: a voxel-wise meta-analysis of voxel based morphometry studies. *Amyotrophic Lateral Sclerosis*.

[34] Cosottini M, Pesaresi I, Piazza S (2012). Structural and functional evaluation of cortical motor areas in Amyotrophic Lateral Sclerosis. *Experimental Neurology*.

[35] Meoded A, Kwan JY, Peters TL (2013). Imaging findings associated with cognitive performance in primary lateral sclerosis and amyotrophic lateral sclerosis. *Dementia and Geriatric Cognitive Disorders Extra*.

[36] Kassubek J, Unrath A, Huppertz HJ (2005). Global brain atrophy and corticospinal tract alterations in ALS, as investigated by voxel-based morphometry of 3-D MRI. *Amyotrophic Lateral Sclerosis*.

[37] Poujois A, Schneider FC, Faillenot I (2012). Brain plasticity in the motor network is correlated with disease progression in amyotrophic lateral sclerosis. *Human Brain Mapping*.

[38] Rule RR, Schuff N, Miller RG, Weiner MW (2010). Gray matter perfusion correlates with disease severity in ALS. *Neurology*.

[39] Block W, Träber F, Flacke S, Jessen F, Pohl C, Schild H (2002). In-vivo proton MR-spectroscopy of the human brain: assessment of N-acetylaspartate (NAA) reduction as a marker for neurodegeneration. *Amino Acids*.

[40] Pohl C, Block W, Karitzky J (2001). Proton magnetic resonance spectroscopy of the motor cortex in 70 patients with amyotrophic lateral sclerosis. *Archives of Neurology*.

[41] Zhang Y, Schuff N, Woolley SC (2011). Progression of white matter degeneration in amyotrophic lateral sclerosis: a diffusion tensor imaging study. *Amyotrophic Lateral Sclerosis*.

[42] Gargiulo-Monachelli GM, Janota F, Bettini M, Shoesmith CL, Strong MJ, Sica REP (2012). Regional spread pattern predicts survival in patients with sporadic amyotrophic lateral sclerosis. *European Journal of Neurology*.

[43] Ravits J, Laurie P, Fan Y, Moore DH (2007). Implications of ALS focality. *Neurology*.

[44] Smith SM, Zhang Y, Jenkinson M (2002). Accurate, robust, and automated longitudinal and cross-sectional brain change analysis. *NeuroImage*.

[45] Smith SM, Jenkinson M, Woolrich MW (2004). Advances in functional and structural MR image analysis and implementation as FSL. *NeuroImage*.

[46] Smith SM (2002). Fast robust automated brain extraction. *Human Brain Mapping*.

[47] Jenkinson M, Smith S (2001). A global optimisation method for robust affine registration of brain images. *Medical Image Analysis*.

[48] Jenkinson M, Bannister P, Brady M, Smith S (2002). Improved optimization for the robust and accurate linear registration and motion correction of brain images. *NeuroImage*.

[49] Zhang Y, Brady M, Smith S (2001). Segmentation of brain MR images through a hidden Markov random field model and the expectation-maximization algorithm. *IEEE Transactions on Medical Imaging*.

[50] Good CD, Johnsrude IS, Ashburner J, Henson RNA, Fristen KJ, Frackowiak RSJ A voxel-based morphometric study of ageing in 465 normal adult human brains.

[51] Ashburner J, Friston KJ (2000). Voxel-based morphometry—the methods. *NeuroImage*.

[52] Andersson JLR, Jenkinson M, Simth S (2007). Non-linear optimisation. *FMRIB*.

[53] Andersson JLR, Jenkinson M, Smith S (2007). Non-linear registration, aka Spatial normalisation. *FMRIB Technical Report*.

[54] Smith SM, Nichols TE (2009). Threshold-free cluster enhancement: addressing problems of smoothing, threshold dependence and localisation in cluster inference. *NeuroImage*.

[55] Cedarbaum JM, Stambler N, Malta E (1999). The ALSFRS-R: a revised ALS functional rating scale that incorporates assessments of respiratory function. *Journal of the Neurological Sciences*.

[56] Ellis C, Suckling J, Amaro E (2001). Volumetric analysis reveals corticospinal tract degeneration and extramotor involvement in ALS. *Neurology*.

[57] Abrahams S, Goldstein LH, Suckling J (2005). Frontotemporal white matter changes in amyotrophic lateral sclerosis. *Journal of Neurology*.

[58] Tedeschi G, Trojsi F, Tessitore A (2012). Interaction between aging and neurodegeneration in amyotrophic lateral sclerosis. *Neurobiology of Aging*.

[59] Rajagopalan V, Liu Z, Allexandre D (2013). Brain white matter shape changes in Amyotrophic Lateral Sclerosis (ALS): a fractal dimension study. *PLoS ONE*.

[60] d' Ambrosio A, Gallo A, Trojsi F (2014). Frontotemporal cortical thinning in amyotrophic lateral sclerosis. *American Journal of Neuroradiology*.

[61] Elamin M, Bede P, Byrne S (2013). Cognitive changes predict functional decline in ALS: a population-based longitudinal study. *Neurology*.

[62] Lillo P, Mioshi E, Burrell JR, Kiernan MC, Hodges JR, Hornberger M (2012). Grey and white matter changes across the amyotrophic lateral sclerosis-frontotemporal dementia continuum. *PLoS ONE*.

[63] Verstraete E, Veldink JH, van den Berg LH, van den Heuvel MP (2014). Structural brain network imaging shows expanding disconnection of the motor system in amyotrophic lateral sclerosis. *Human Brain Mapping*.

[64] Hanakawa T, Immisch I, Toma K, Dimyan MA, Van Gelderen P, Hallett M (2003). Functional properties of brain areas associated with motor execution and imagery. *Journal of Neurophysiology*.

[65] Turner MR, Barnwell J, Al-Chalabi A, Eisen A (2012). Young-onset amyotrophic lateral sclerosis: historical and other observations. *Brain*.

[66] Turner MR, Wicks P, Brownstein CA (2011). Concordance between site of onset and limb dominance in amyotrophic lateral sclerosis. *Journal of Neurology, Neurosurgery and Psychiatry*.

[67] Agosta F, Gorno-Tempini ML, Pagani E (2009). Longitudinal assessment of grey matter contraction in amyotrophic lateral sclerosis: a tensor based morphometry study. *Amyotrophic Lateral Sclerosis*.

